# Forkhead box M1 recruits FoxP3^+^ Treg cells to induce immune escape in hilar cholangiocarcinoma

**DOI:** 10.1002/iid3.727

**Published:** 2022-10-26

**Authors:** Kai Ma, Zhaowei Sun, Xueliang Li, Jingyun Guo, Qinlei Wang, Mujian Teng

**Affiliations:** ^1^ Department of General Surgery, Shandong Qianfoshan Hospital, Cheeloo College of Medicine Shandong University Jinan Shandong Province P.R. China; ^2^ Department of Hepatopancreatobiliary Surgery The Affiliated Hospital of Qingdao University Qingdao Shandong Province P.R. China

**Keywords:** CD8^+^T cells, FoxM1, FoxP3, Hilar cholangiocarcinoma, immune escape, Treg cells

## Abstract

**Objective:**

Hilar cholangiocarcinoma (HCCA) is a malignancy related to chronic biliary tract inflammation. Tumor immune escape is a necessary process of tumorigenesis. Forkhead box M1 (FoxM1) could affect the progression of various carcinomas. This study attempted to elaborate on the mechanism of FoxM1 in HCCA immune escape.

**Methods:**

HCCA cell lines were collected to measure the expression of FoxM1 and FoxP3. CD8^+^ T cells were extracted to establish the co‐culture system with HCCA cells and Treg cells. pcDNA3.1‐FoxM1 or si‐FoxP3 was transfected into HCCA cells in the co‐culture system. HCCA cell viability, mobility, and invasiveness as well as levels of transforming growth factor (TGF)‐β and interleukin (IL)‐6 were evaluated. The binding relation between FoxM1 and FoxP3 promoter was verified. HCCA cells with pcDNA3.1‐FoxM1 were subcutaneously injected into mice to establish the xenograft mouse models.

**Results:**

FoxM1 and FoxP3 were overexpressed in HCCA cells. The co‐culture of CD8^+^ T and HCCA cells inhibited HCCA cell activity and Treg cells limited CD8^+^ T killing. FoxM1 overexpression strengthened the inhibiting role of Treg cells in CD8^+^ T killing, upregulated TGF‐β and IL‐6 levels, and encouraged HCCA immune escape. FoxM1 bound to the FoxP3 promoter region to promote FoxP3 transcription. Silencing of FoxP3 neutralized the promoting role of FoxM1 overexpression in Treg cell immunosuppression and HCCA cell immune escape. FoxM1 aggravated tumor development, upregulated FoxP3 expression, increased Treg cells, and reduced CD8^+^ T cells.

**Conclusion:**

FoxM1 bound to the FoxP3 promoter region to promote FoxP3 transcription and recruited FoxP3^+^ Treg cells, thereby inducing HCCA immune escape.

## Graphical abstract

Forkhead box M1 bound to the FoxP3 promoter region to promote FoxP3 transcription and recruit FoxP3+ Treg cells, thereby inducing Hilar cholangiocarcinoma immune escape.

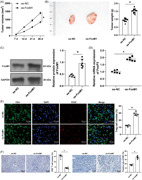

## 1 INTRODUCTION

Hilar cholangiocarcinoma (HCCA) is a liability in the network of veins, arteries, and bile ducts, retarding hepatic confluence and disrupting biliary functions, and leading to dangers for distant organs, vascular system, and nervous tissues.^1^ HCCA is associated with hepatolithiasis, primary sclerosing cholangitis, and hepatopathy, with clinical symptoms including stomachache, weight reduction, pruritus, and icterus.^2^ For now, the available options for alleviating HCCA are chemoradiotherapy, excision of tumor, organ transplantation, and removal of the extrahepatic bile duct.^3^ However, due to retarded diagnosis and tumor metastasis, resection of advanced HCCA remains a clinical challenge.^4^ Worse still, HCCA immune escape developing in the malignant microenvironment, in turn, accelerates cancer growth and neutralizes therapeutic efficiency.^5^ Tumor‐related cytokines are necessary for tumorigenesis and have therapeutic significance in HCCA.^6^, ^7^ Hence, this paper is proposed to probe the possible cytokines involved in HCCA immune escape.

Forkhead box O (FOXO) family participates in cancer development by regulating cell viability, metabolism, metastasis, senescence, self‐renewal, cancer immunity, and inflammatory responses.^8^ As an important member of the FOXO family, Forkhead box M1 (FoxM1) affects cancer cell biological behaviors by manipulating the transcription of its target genes. FoxM1 is strongly expressed in a variety of neoplasms and is related to poor clinical outcomes.^9^ Importantly, FoxM1 ablation discourages CCA cell activity and improves overall survival rates.^10^ FoxP3, another member of the FOXO family, is a major player in the immune system, and it consolidates cancer cell immunosuppression to anticancer drugs or cells.^11^ FOXP3 secrets immune‐suppressive cytokines to catalyze immune escape and predicts poor prognosis in CCA.^12^ But the mechanism of FoxM1 and FoxP3 in the immune escape of HCCA remains unknown.

Regulatory T (Treg) cells are featured by an increasing amount of FOXP3 content and are recruited in the tumor microenvironment to eradicate obstacles to tumor growth.^13^ Furthermore, Treg cells balance microenvironment homeostasis, stimulate inflammatory reactions, accelerate cell cargo, and strengthen immune escape in cancers.^14^ CD8^+^ T cells, also known as cytotoxic T‐lymphocytes, have the highest killing efficacy in immunity, and Treg cells regulate CD8^+^ T cell activity to control cancer cell resistance and immune escape.^15^ CD8^+^ T killing mechanism could accelerate cell death and enhance cell chemosensitivity to reduce CCA malignancy.^16^ Taking the above‐mentioned evidence into account, we hypothesize that FOXM1 might mediate HCCA immune escape via the manipulation of FoxP3^+^ Treg and CD8^+^ T killing. Therefore, the co‐culture system of HCCA cells, CD8^+^ T, and Treg cells was established to elaborate the effects of FoxM1 in recruiting Treg cells to reduce CD8^+^ T cells and mediating the immune escape of HCCA.

## 2 MATERIALS AND METHODS

### 2.1 Ethics‐statement

This study was approved and supervised by the ethics committee of Shandong Qianfoshan Hospital. The protocol complied with the *Guide for the Care and Use of Laboratory Animals* provisions for the administration and usage of laboratory animals,^17^ ARRIVE checklist see the supplementary file. Great efforts were made to reduce both the number of animals and their suffering.

### 2.2 Cell culture

Human HCCA cell lines (QBC939 and RBE) and human intrahepatic bile duct epithelial cells (HIBECs) (all from Shanghai YaJi Biotechnology Co, Ltd) were cultured at Roswell Park Memorial Institute (RPMI)‐1640 medium (SLM‐240; Sigma) with 10% fetal bovine serum (FBS) at 37°C with 5% CO_2_. HCCA cells were confirmed negative for mycoplasma infection using the mycoplasma quantitative polymerase chain reaction (PCR) assay kit (MP0035; Sigma).

Overexpression of FoxM1 vector (pcDNA3.1‐FoxM1), negative control of FoxM1 vector (pcDNA3.1‐NC), small interfering (si) RNAs of FoxP3 (si‐FoxP3‐1 and si‐FoxP3‐2), and si‐NC (all from Genechem) were transfected into HCCA cells following the instructions of Lipofectamine 2000 (11668019; Invitrogen Inc).

### 2.3 Separation and purification of CD8^+^ T cells and CD4^+^ CD25^+^ Treg cells

Human peripheral blood (PB) was collected from healthy volunteers while human PB mononuclear cells (PBMCs) were isolated using Histopaque‐1077 (10771; Sigma). Human CD8^+^ T cells were extracted using the MagniSort human CD8 positive selection kit (8802‐6832‐74; Invitrogen), resuspended in phosphate buffer saline (PBS), and then cultured with 25 μl Human T‐activator CD3/CD8 T cell stimulator (to activate and expand CD8^+^ T cells) (11161D; R&D Systems) and 20 U/ml interleukin (IL)‐2 (to secure the growth of CD8^+^ T cells) at 37°C for 48 h. Separated cells were incubated with Alexa Fluor® 488‐coupled anti‐CD8 antibody (1:500; ab237364) and underwent flow cytometry for separation and purification.

CD4^+^ T cells were removed from PBMCs using the anti‐CD4 magnetic beads (11331D; Invitrogen) and incubated in the complete RPMI‐1640 medium containing 20 IU/ml IL‐2. Naïve CD4^+^ T cells were purified using the CD4^+^ T cell purification kit (130‐094‐131; Miltenyi Biotec Technology & Trading). To induce the differentiation of CD4^+^ T cells into Treg cells, CD4^+^ T cells were challenged by anti‐CD3 monoclonal antibody (mAb) (1:500; ab16669; Abcam) and anti‐CD28 mAb (1:500; ab243228; Abcam) containing human transforming growth factor (TGF)‐β1 (2.5 ng/ml, 240‐B; R&D Systems Inc.) and mouse IL‐2 (10 ng/ml; 402‐ML; R&D Systems), respectively for 3 days. Separated cells were incubated with fluorescein isothiocyanate‐coupled anti‐CD4 antibody (1:500; ab59474; Abcam) and allophycocyanin‐coupled anti‐CD25 antibody (1:500; ab267381; ab2673810), followed by flow cytometry for separation and purification.

### 2.4 Co‐culture system of HCCA cells and CD8^+^ T cells

HCCA cells were co‐cultured with CD8^+^ T cells in 6‐well Transwell co‐culture plates (0.4 μm polyester film). According to the previous study,^18^ CD8^+^ T cells of different concentrations (1 × 10^4^, 5 × 10^4^, 1 × 10^5^, 2 × 10^5^, and 5 × 10^5^ cells/well) were seeded in the apical layer, while HCCA cells (2 × 10^4^ cells/well) were seeded in the basolateral layer, from which HCCA cells were then appointed for cell counting kit (CCK)‐8 assay to detect cell activity and screen the optimal concentration of CD8^+^ T cells. Subsequently, a co‐culture system of HCCA cells, CD8^+^ T cells, and Treg cells was established. CD8^+^ T cells (2 × 10^5^ cells/well) and Treg cells (2 × 10^5^ cells/well) were seeded in the apical layer of the Transwell co‐culture plate at the proportion of 1: 1, while HCCA cells (2 × 10^4^ cells/well) were seeded in the basolateral layer. After 48 h of co‐culture, HCCA cells were then applied for CCK‐8 assay to evaluate cell activity. Cells were cultured in RPMI‐1640 medium with 10% FBS and 5% CO_2_ at 37°C.

### 2.5 CCK‐8 assay

The CCK‐8 assay kits (C0037; Beyotime Biotechnology) were appointed to assess HCCA cell activity. In brief, HCCA cells (2 × 10^4^ cells/well) were seeded in 96‐well plates in RPMI‐1640 medium with 10% FBS, with 10 μl CCK‐8 reagent supplemented into each well, and then subjected to the culture at 37°C for 1 h. The optical density value at the wavelength of 450 nm was evaluated by a standard microplate reader (Bio‐Rad 680; Bio‐Rad).

### 2.6 Enzyme‐linked immunosorbent assay (ELISA)

TGF‐β (KE1373) and IL‐6 (KE1368) levels were detected using the ELISA kits (Immunoway) following the manufacturers' instructions. After cocultured cells were centrifuged at 1000 g, the supernatant from each group was obtained and reacted with 100 μl enzyme‐linked reagent at 37°C for 30 min. Next, 100 μl horseradish peroxidase (HRP) substrate solution was supplemented to each well for 20 min incubation at 37°C. Finally, 50 μl termination solution was supplemented into each well and the optical density value at the wavelength of 450 nm was assessed within 20 min using a standard microplate reader.

### 2.7 Reverse transcription‐quantitative PCR (RT‐qPCR)

The total RNA was removed from HCCA cells utilizing the TRIzol reagent (15596018; Invitrogen) and reverse‐transcribed into cDNA via the PrimeScript^TM^ RT kits (6110A; Takara Bio Inc). synergy brand Green served as the detection fluorophore of qPCR, which was carried out on the 7900 HT Fast RT‐PCR system (Applied Biosystems, Inc). The RT‐qPCR primers were seen in Table 1. The fold change values of the target genes were standardized to glyceraldehyde‐3‐phosphate dehydrogenase (GAPDH). The relative expressions of genes were calculated employing the $2^{‐∆∆C_{t}}$ method.

**Table 1:** Primer sequence of RT‐qPCR

Gene	Forward Primer (5′‐3)	Reverse Primer (5′‐3′)
FoxM1	AGATTCATAATGAAAACT	GGCAGGGCTCTACTGTAG
Foxp3	AAGGACCCGATGCCCAAC	ATCTTGAGGTCAGGGGCC
GAPDH	CTCAACTACATGGTTTAC	CCAGGGGTCTTACTCCTT
Foxp3 promoter	GATCTTGGCCACCAGATTT	GGTCAGCATGGTAGACCAG

Abbreviations: F, forward; FoxM1, forkhead box M1; Foxp3, forkhead box P3; GAPDH, glyceraldehyde‐3‐phosphate dehydrogenase; R, reverse; RT‐qPCR, reverse transcription‐quantitative polymerase chain reaction.

### 2.8 Western blot analysis

The total protein was extracted from HCCA cells or homogenate of tumor tissue using radio‐immunoprecipitation assay cell lysis buffer (20–188, Sigma), with protein concentration measured using the bicinchoninic acid protein assay kits (23235; Thermo Fisher Scientific). Protein samples were separated through 10% sodium dodecyl sulfate‐polyacrylamide gel electrophoresis and then transferred onto polyvinylidene fluoride membranes, and blocked with bovine serum albumin, and incubated with anti‐FoxM1 (1:1000; ab207298; Abcam Inc) and anti‐GAPDH (1:2500; ab8245; Abcam) at 4°C overnight. Next, the membranes were cultivated with HRP‐conjugated immunoglobulin G (IgG; 1: 2000; ab6721; Abcam) for 2 h. The membranes were mixed with luminol‐based chemiluminescence substrate and the protein bands were analyzed by the Image J software (Bio‐Rad Laboratories), with GAPDH as the internal reference.

### 2.9 Transwell assays

HCCA cells were obtained from the system where HCCA cells, CD8^+^ T, and Treg cells were cocultured. Cell invasion was determined through Transwell assays.^19^ HCCA cells were cultured in serum‐free RPMI‐1640 medium and placed on the filtering membrane (8 μm‐well) of the Transwell insert. RPMI‐1640 medium containing 10% FBS was placed in the basolateral chamber. After 24 h of growth, cells on the filter membrane migrated into the basolateral chamber. Next, cells were fixed using 4% paraformaldehyde/sucrose solution for 10–15 min, with 0.2% crystal violet added for staining, and the stained cells were observed and counted under an inverted microscope (Olympus). For the invasion experiment, the apical chamber was pre‐coated with substrate before cell incubation. After 24 h of culture, cells in the basolateral chamber passed through the filter membrane to invade cells in the apical chamber.

### 2.10 Chromatin immunoprecipitation (ChIP) assay

The ChIP assay was performed according to the instructions of the EZ ChIP assay kit (17‐371; Millipore). Cells were lysed using radioimmunoprecipitation assay buffer (Sigma) and fixed with 1% methanal to crosslink DNA and protein, followed by ultrasonic treatment to fragment DNA. According to the instructions of assay kits, chromatins were incubated with antibodies against FoxM1 (20459; Cell Signaling Technology) or IgG (ab172730; Abcam) at 4°C overnight. Chromatins were purified using the DNA purification kit (17290; Intron Biotechnology), followed by RT‐qPCR. Primer sequences are shown in Table 1.

### 2.11 Dual‐luciferase reporter gene assay

The binding site of FoxM1 and the FoxP3 promoter was predicted through the JASPAR online database (https://jaspar.genereg.net/). Luciferase reporter gene plasmids containing wild type (WT) and mutant type (MUT) sequences of the FoxP3 promoter binding to FoxM1 were co‐transfected into HCCA cells with pcDNA3.1‐FoxM1 or pcDNA3.1‐NC using Lipofectamine 2000 according to the manufacturers' instructions. After 48 h of incubation, luciferase activity was assessed with the dual‐luciferase reporter gene assay system (Promega Biotech Co, Ltd). All steps were repeated three times and the mean value was calculated for statistical analysis.

### 2.12 Xenografts tumors in nude mice

Twenty‐four BALB/c mice (4–6 weeks) (Shanghai SLAC Laboratory Animal Co, Ltd, 2017‐0008] were raised in the pathogen‐free environment under 12 h light‐dark cycles at constant temperature (25 ± 2°C) and humidity (70%), with available food and water. All animals were numbered by weight and grouped according to the random number method. HCCA cells were infected with the lentivirus‐packaged plasmids overexpression FoxM1 (oe‐FoxM1) and oe‐NC (both from Genechem) at a multiplicity of infection of 30 and treated with puromycin (5 μg/ml) to screen stable HCCA cells overexpressing FoxM1. Subsequently, RBE cells (3 × 10^6^/mouse) were subcutaneously injected into the posterolateral side of mice (*N* = 12/group). Tumor volume was determined every 7 day according to the formula (length ×l width^2^/2). The health and behaviors of all animals were monitored daily, and nude mice were euthanized when the following situations (humane endpoints) occurred: weight loss >15% of the nude mouse body weight, or the animal suffered from tumor load, or the maximum diameter of the tumor exceeded 1.5 cm. No animals died in the midway of the experiments. After 4 weeks, all mice were euthanatized with 1% sodium pentobarbital (150 mg/kg; Sigma) through intraperitoneal injection. Tumors were isolated for weighing. Next, the tumors of 6 mice were randomly selected for RT‐qPCR or Western blot assay, and the tumors of the other 6 mice were employed for immunohistochemistry (IHC).

### 2.13 Dual enzyme‐labeled immunofluorescence

Mouse tumor sections were fixed using formalin, paraffin‐embedded, and then cultured with general heat‐induced epitope retrieval antigen extraction reagent (Abcam) at 110°C and high pressure for 10 min. When cooling to 60°C, the sections were rinsed using 10 mmol/L Tris‐hydrochloride (pH 7.4) buffered saline for 5 min and stained with primary antibody for 60 min. Then, the sections were washed twice (5 min/time) and reacted with peroxidase polymer anti‐rabbit IgG for 30 min. Subsequently, the sections were washed twice (5 min/time) and the signals in the sections were visualized through tyramine signal amplification and the OPAL system. Sections were then heated with 10 mmol/L sodium citrate buffer (pH 6.0) at 95°C for 10 min to discard the binding antibody, followed by the next primary antibody staining. The subsequent staining was performed identically. Cell nuclei were stained using 4′, 6‐diamidino‐2‐phenylindole. The following antibodies were applied in this experiment: rabbit anti‐CD4 (1:500; ab248580; Abcam), mouse anti‐CD25 (1:50; ab9496; Abcam), goat anti‐rabbit secondary anti‐IgG (1:2000; ab150077; Abcam) and rabbit anti‐mouse IgG (1:1000; ab150115; Abcam). Results were analyzed by two researchers who were uninformed of experiments using Image J software (National Institutes of Health).

### 2.14 IHC

IHC staining was performed on mouse tumor Section (4 μm). Briefly, tumor tissues were paraffin‐embedded, deparaffinized, rehydrated using graded alcohol, and washed twice in PBS (10 min/time). Then, sections were incubated with rabbit anti‐CD8 (0.25 µg/ml; ab237709; Abcam) and rabbit anti‐Ki67 (0.1 µg/ml; ab15580; Abcam) overnight, followed by incubation with HRP‐conjugated goat anti‐rabbit IgG (1:2000; ab150077; Abcam) at 37°C for 30 min. Sections were stained with 3 '3‐diaminobenzidine working solution for 3 min, rinsed using water for 10 min, and counterstained with hematoxylin. After the sections were repeatedly washed in water for 10 min, they were dehydrated and observed using a microscope (Olympus). Results were analyzed by two researchers who were uninformed of experiments using Image J software.

### 2.15 Statistical analysis

GraphPad Prism 8.0 software (GraphPad Software Inc) was employed for data analysis and graphing. Measurement data were expressed as mean ± standard deviation. The independent *t*‐test was employed for comparison analysis between two groups and one‐way or two‐way analysis of variance (ANOVA) was employed for comparison analysis among various groups, and Tukey's post‐hoc test was used for posttest of data. The *p* value was calculated through a two‐tailed test and a value of *p* < .05 indicated a significant difference.

## 3 RESULTS

### 3.1 Treg cells suppress CD8^+^ T killing and enhance HCCA cell proliferation

**Figure 1:**
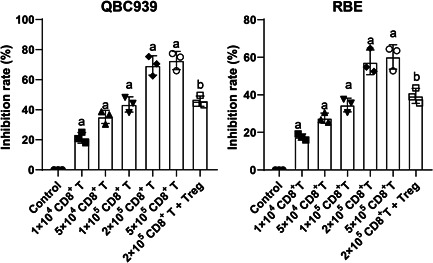
Treg cells suppress CD8^+^ T killing and enhance HCCA cell proliferation. Co‐culture system of CD8^+^ T cells at different concentration gradients and HCCA cells was established. The optimal concentration of CD8^+^ T cells was selected through CCK‐8 assay, and then Treg cells at the same concentration were added to the co‐culture system, and the inhibition rate of CD8^+^ T cells on HCCA cells was measured by CCK‐8 assay. Independent experiments were repeated 3 times. The results were expressed as mean ± standard deviation. One‐way analysis of variance was appointed to analyze the data. Tukey's post‐hoc test was applied for the post hoc test. a, compared with the control group, *p* < .05; b, compared with the 2 × 10^5^ CD8^+^ T cell group, *p* < .05. HCCA, Hilar cholangiocarcinoma.

To figure out the immune escape mechanism of HCCA cells, CD8^+^ T cells were extracted from human PBMCs and co‐cultured with HCCA cells (QBC939 and RBE). HCCA cell activity was measured by CCK‐8 assay, which found that CD8^+^ T cells reduced HCCA cell activity (*p* < .05, Figure 1). In the co‐culture system, when CD8^+^ T cell concentration reached 2.0 × 10^5^/well, they could kill HCCA cells. The same amount of Treg cells was supplemented into the co‐culture system of CD8^+^ T cells (2.0 × 10^5^/well) and HCCA cells, after which we noticed that HCCA cell activity was significantly promoted (*p* < .05, Figure 1). Collectively, Treg cells could suppress CD8^+^ T killing and enhance HCCA cell proliferation.

### 3.2 FoxM1 is robustly expressed in human HCCA cells

**Figure 2:**
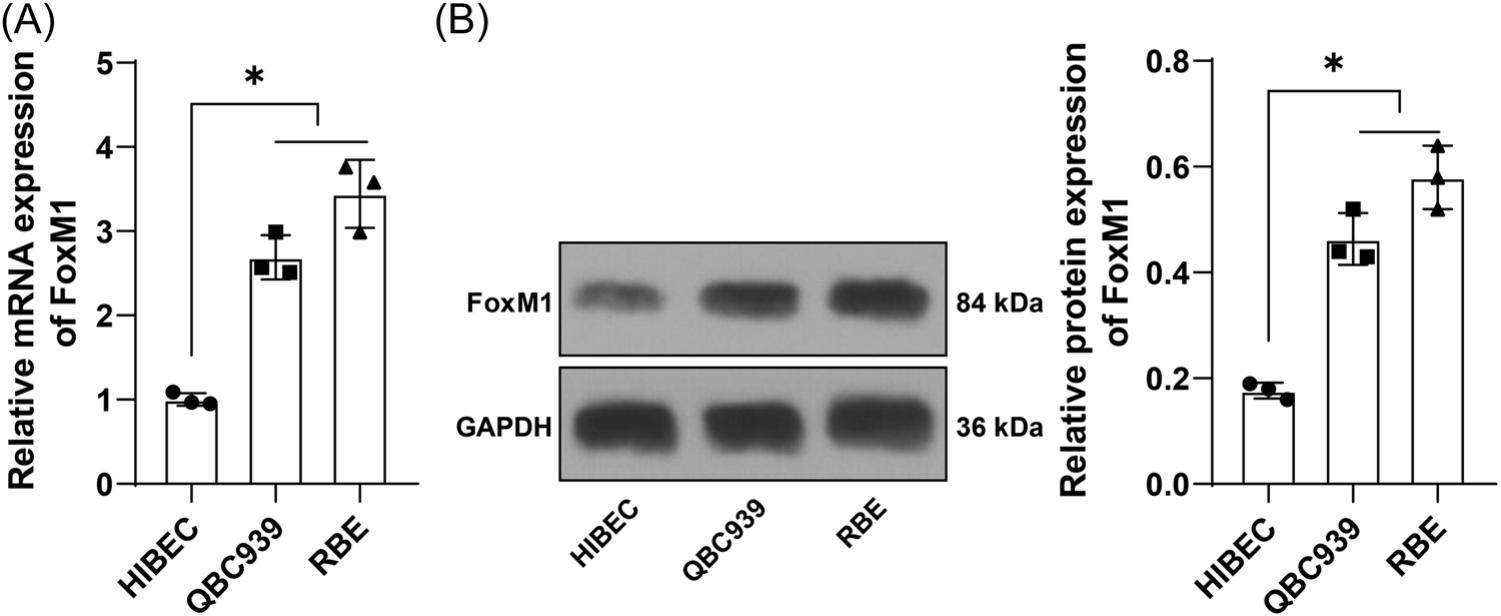
FoxM1 is robustly expressed in human HCCA cells. (A) and (B) FoxM1 expression in human HCCA cells was determined by reverse transcription‐quantitative polymerase chain reaction (A) and Western blot analysis (B). Independent experiments were repeated three times. The results were expressed as mean ± standard deviation. One‐way analysis of variance was used to analyze the data in (A) and (B). Tukey's post‐hoc test was applied for the post‐hoc test. *p < .05. FoxM1, Forkhead box M1; HCCA, Hilar cholangiocarcinoma.

Then the molecular mechanism of the immune escape of HCCA cells was explored. It was reported that FoxM1 was highly expressed in gallbladder carcinoma, and its overexpression encouraged multiple carcinomas and predicted a frustrating prognosis.^20^ Therefore, we hypothesized that FoxM1 may be correlated with the immune escape of HCCA cells. FoxM1 expression in human HCCA cells was determined by RT‐qPCR and Western blot analysis, which revealed that compared with HIBECs, FoxM1 was overexpressed in HCCA cells (*p* < .05, Figure 2A,B).

### 3.3 HCCA cells with FoxM1 overexpression sabotage CD8^+^ T killing on HCCA cells, thereby facilitating HCCA cell immune escape

In the following experiments, FoxM1 was overexpressed to elucidate the role of FoxM1 in HCCA cell immune escape. pcDNA3.1‐FoxM1 was transfected into HCCA cells to overexpress FoxM1 (*p* < .05, Figure 3A,B), and the results indicated that there was no evident difference in CD8^+^ T killing on HCCA cells between the coculture system and the oe‐FoxM1 group (*p* > .05, Figure 3C), while with the addition of Treg cells, FoxM1 overexpression attenuated CD8^+^ T killing on HCCA cells (*p* < .05, Figure 3D). The supernatant of the coculture system of HCCA cells, CD8^+^ T cells, and Treg cells was tested by ELISA, which revealed that FoxM1 overexpression led to increased levels of TGF‐β and IL‐6 (*p* < .05, Figure 3E). The results of Transwell assays unveiled that FoxM1 overexpression enhanced HCCA cell migration and invasion (*p* < .05, Figure 3F,G). The above findings suggested that FoxM1 overexpression accelerated HCCA cell immune escape by promoting Treg cell immunosuppression on CD8^+^ T cells.

Figure 3HCCA cells with FoxM1 overexpression sabotage CD8^+^ T killing on HCCA cells, thereby improving HCCA cell immune escape. pcDNA3.1‐FoxM1 was transfected into HCCA cells, with pcDNA3.1‐NC transfection as the control. (A) and (B), FoxM1 expression was detected by reverse transcription‐quantitative polymerase chain reaction (A) and Western blot analysis (B). Then FoxM1 was co‐cultured with CD8^+^ T cells. (C) CD8^+^ T cell immunosuppression on HCCA cells was tested by CCK‐8 assay. The co‐culture system of HCCA cells, CD8^+^ T cells, and Treg cells was established. (D) CD8^+^ T cell immunosuppression on HCCA cells was tested by CCK‐8 assay. (E) Levels of TGF‐β and IL‐6 in the co‐culture system were assessed by ELISA. (F) and (G), HCCA cell migration (F) and invasion (G) were evaluated by Transwell assays. Independent experiments were repeated three times. The results were expressed as mean ± standard deviation. Two‐way analysis of variance (ANOVA) was appointed to analyze the data in (E), and one‐way ANOVA was used to analyze the data in (A), (B), (C), (D), (F), and (G). Tukey's post‐hoc test was applied for the post hoc test. *p < .05. CCK, cell counting kit; E, enzyme‐linked immunosorbent assay; FoxM1, Forkhead box M1; HCCA, Hilar cholangiocarcinoma; IL, interleukin; TGF, transforming growth factor.

**Figure:**
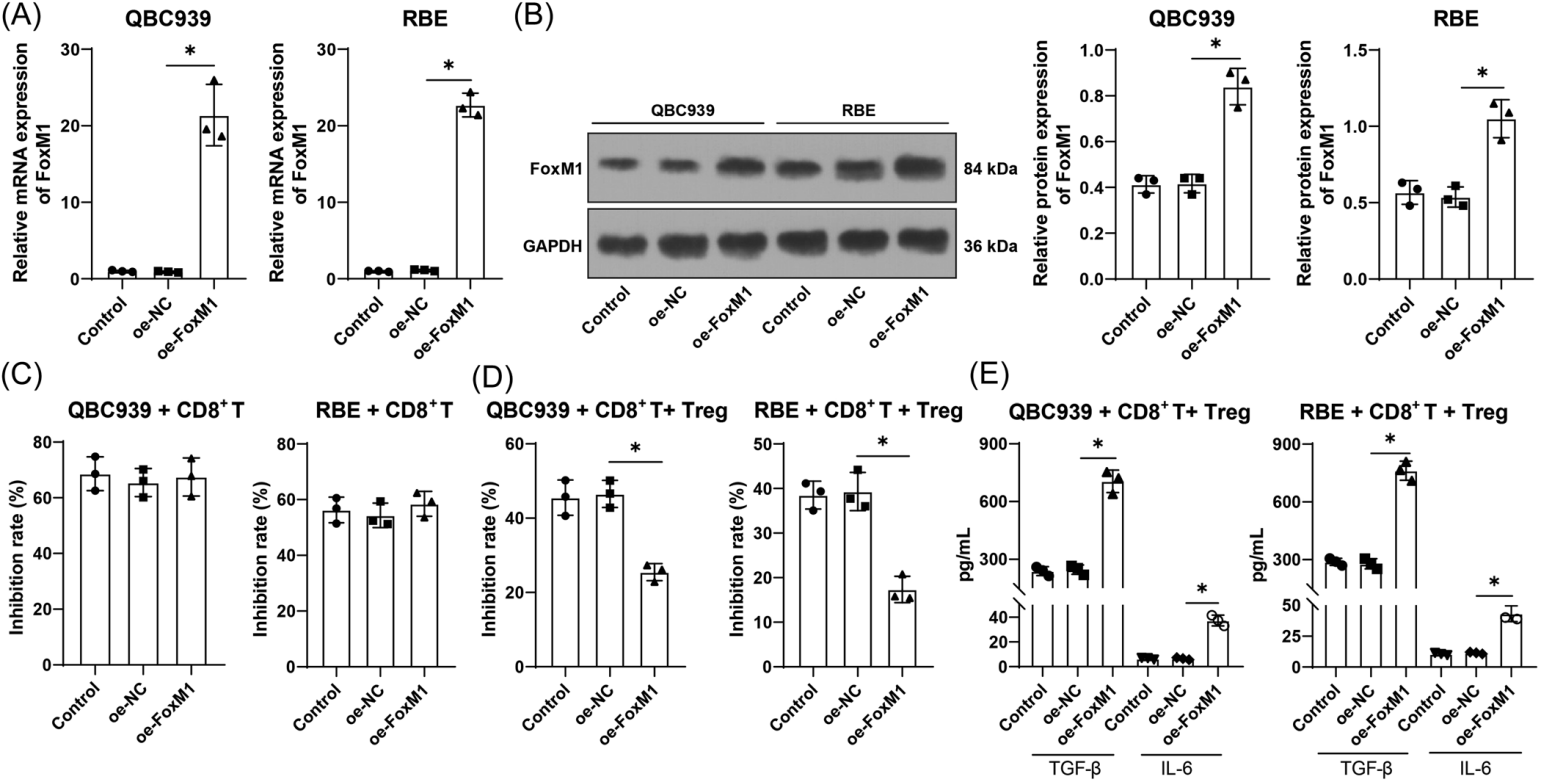

**Figure:**
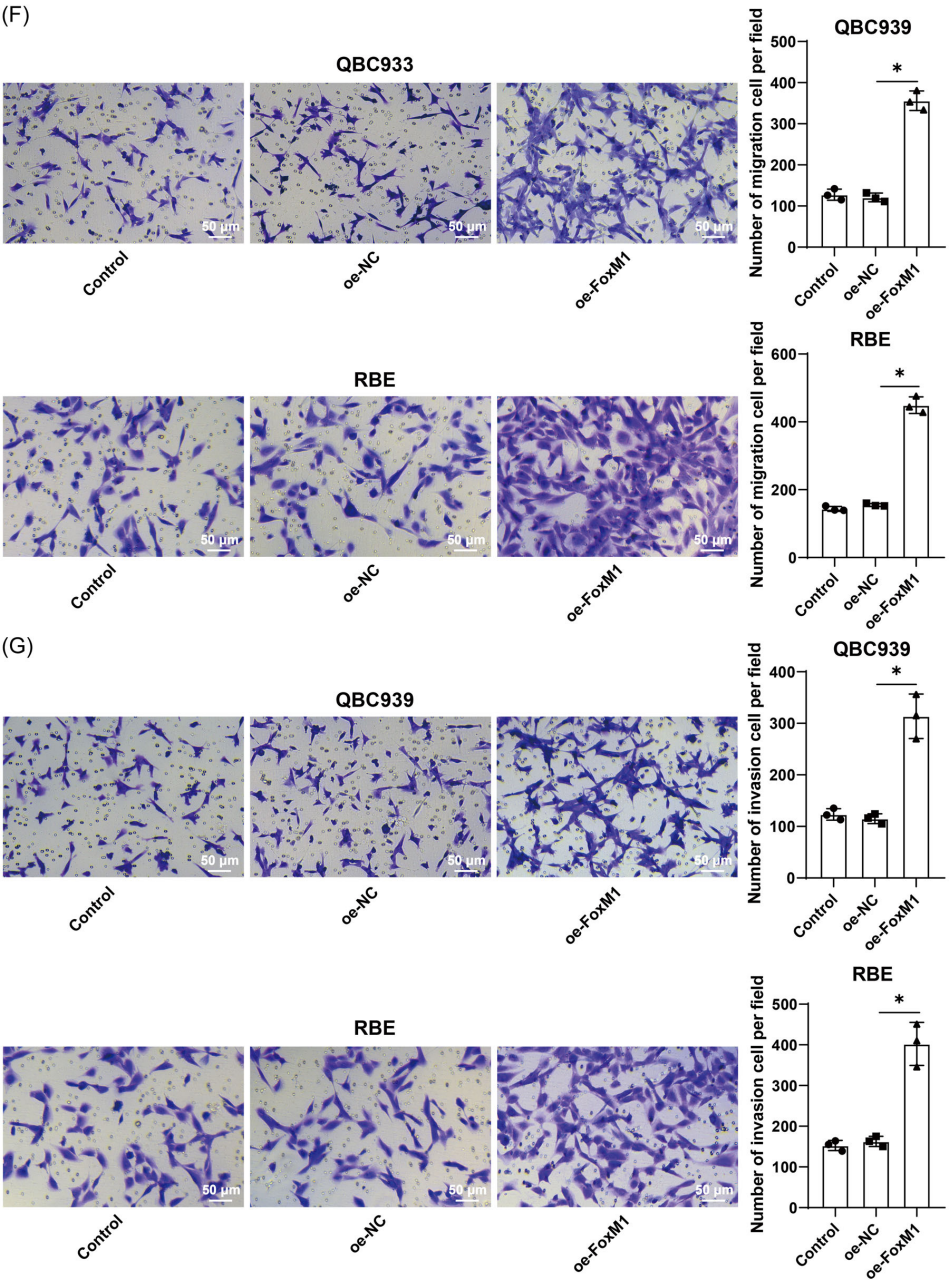

### 3.4 FoxM1 could bind to the FoxP3 promoter region to promote FoxP3 transcription

**Figure 4:**
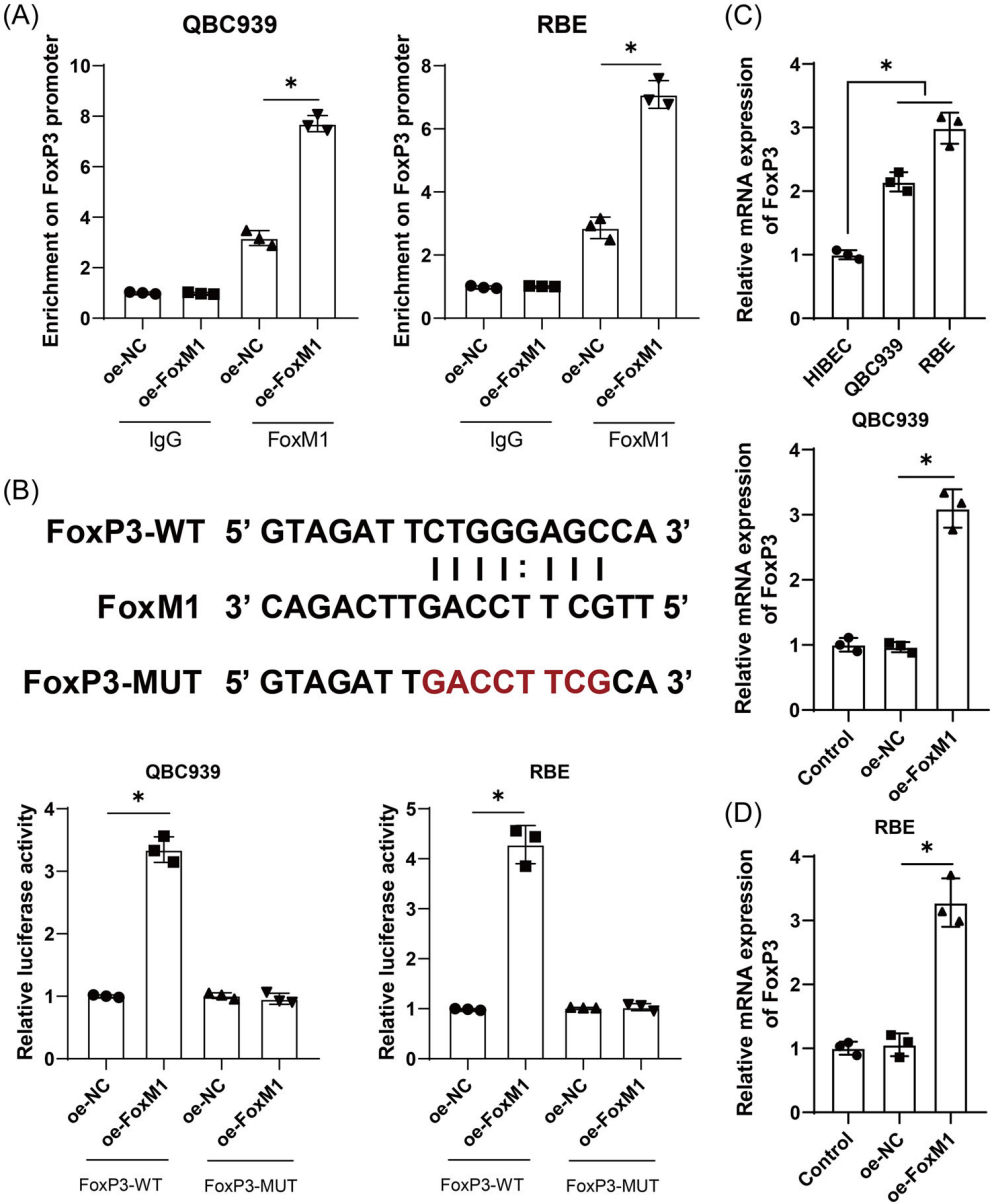
FoxM1 could bind to the FoxP3 promoter region to promote FoxP3 transcription. (A) The enrichment of FoxM1 on the FoxP3 promoter was analyzed by the ChIP assay. (B) The binding relation between FoxM1 and the FoxP3 promoter was detected by dual‐luciferase reporter gene assay. (C) and (D), FoxP3 mRNA level in HCCA cells was assessed by RT‐qPCR. Independent experiments were repeated three times. The results were expressed as mean ± standard deviation. Two‐way analysis of variance (ANOVA) was used to analyze the data in panels A and B, and one‐way ANOVA was used to analyze the data in (C) and (D). Tukey's post‐hoc test was applied for the post‐hoc test. *p < .05. FoxM1, Forkhead box M1; RT‐qPCR, reverse transcription‐quantitative polymerase chain reaction.

FoxP3 is an important regulatory element in Treg cell viability and growth and a well‐recognized marker of Treg cells. FoxM1 could induce immunosuppression by upregulating FoxP3^+^ Treg cells.^21^ Collectively, we hypothesized that FoxM1 might mediate FoxP3 expression to modulate Treg cell immunosuppression. The ChIP assay showed that FoxM1 was abundantly enriched in the FoxP3 promoter (*p* < .05, Figure 4A). Then, oe‐FoxM1 and FoxP3 promoter luciferase reporter gene plasmids were co‐transfected into HCCA cells, and the results indicated that FoxM1 overexpression encouraged the luciferase activity of the FoxP3‐WT group but rendered few changes to the FoxP3‐MUT group (*p* < .05, Figure 4B). Then FoxP3 expression in HCCA cells was examined, and we observed that FoxP3 expression was higher in HCCA cells than that in HIBECs (*p* < .05, Figure 4C), and it could be upregulated by FoxM1 overexpression (*p* < .05, Figure 4D). All in all, FoxM1 could bind to the FoxP3 promoter region to promote FoxP3 transcription.

### 3.5 Silencing of FoxP3 neutralizes the promotive role of FoxM1 overexpression in HCCA cell immune escape

**Figure 5:**
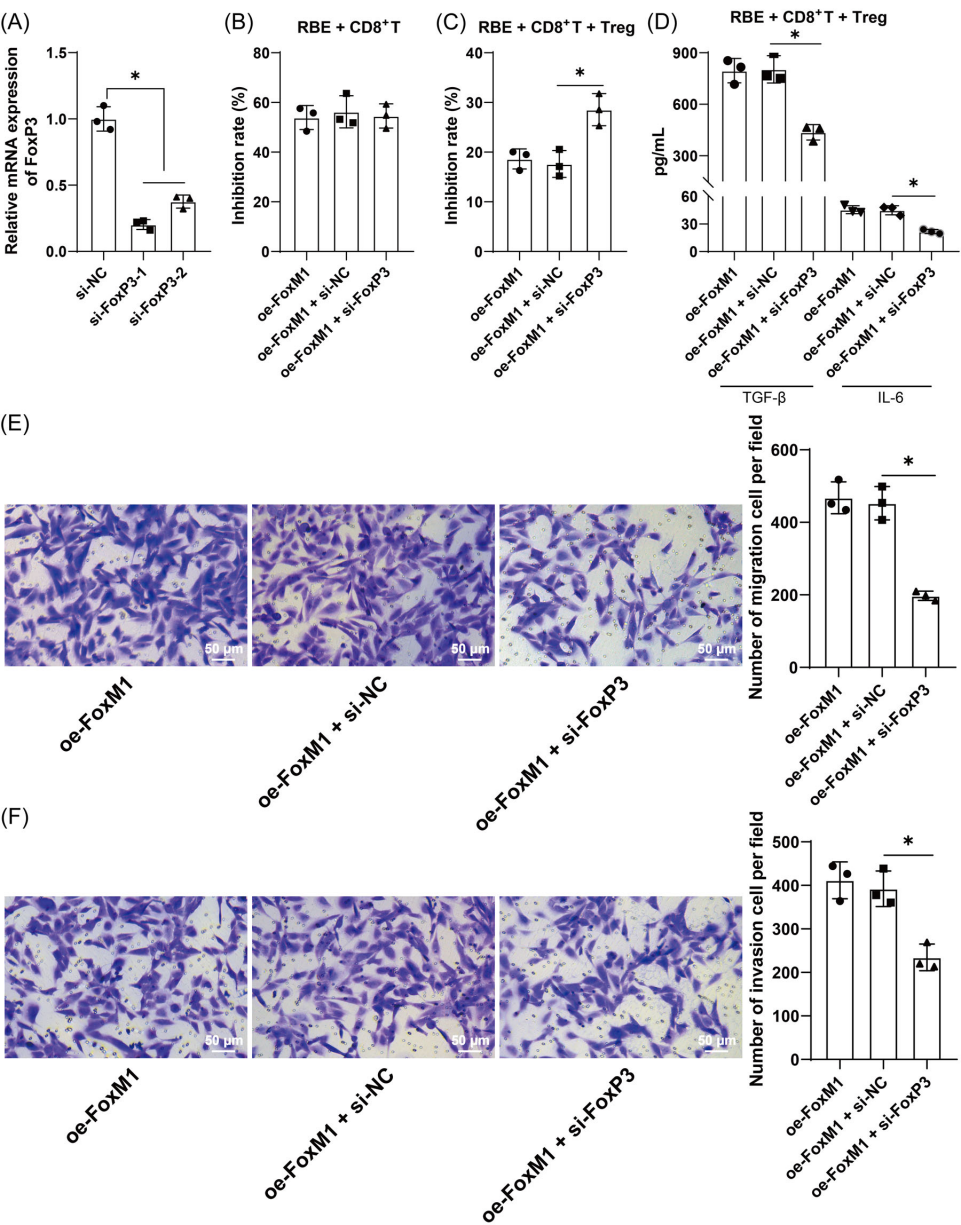
Silencing of FoxP3 neutralizes the promotive role of FoxM1 overexpression in HCCA cell immune escape. si‐FoxP3‐1 and si‐FoxP3‐2 were transfected into RBE cells. (A) Transfection efficacy was detected by RT‐qPCR. si‐FoxP3‐1 was transfected into RBE cells to conduct combined experiments with oe‐FoxM1 and then co‐cultured with CD8^+^ T cells. (B) CD8^+^ T cell immunosuppression on RBE cells was tested by CCK‐8 assay. The co‐culture system of HCCA cells, CD8^+^ T cells, and Treg cells was established. (D) Levels of TGF‐β and IL‐6 in the co‐culture system were assessed by ELISA. (E) and (F) RBE cell migration (E) and invasion (F) were evaluated by Transwell assays. Independent experiments were repeated three times. The results were presented as mean ± standard deviation. Two‐way analysis of variance (ANOVA) was used to analyze the data in (D), and one‐way ANOVA was used to analyze the data in (A), (B), (C), (E), and (F). Tukey's post‐hoc test was applied for the post hoc test. *p < .05. FoxM1, Forkhead box M1.

To further confirm that FoxM1 can modulate FoxP3 expression to influence Treg cell immunosuppression, si‐FoxP3‐1 and FoxP3‐2 were transfected into RBE cells, which had higher FoxM1 expression, to silence FoxP3 expression (*p* < .05, Figure 5A). Subsequently, oe‐FoxM1 and si‐FoxP3‐1, the ones with better silencing efficacy, were co‐transfected into RBE cells to be cocultured with CD8^+^ T cells. The results showed that the silencing of FoxP3 did not bring significant change to RBE cell activity (*p* > .05, Figure 5B), while with the addition of Treg cells, the silencing of FoxP3 suppressed RBE cell activity (*p* < .05, Figure 5C). The supernatant of the co‐culture system was tested by ELISA, which suggested that the silencing of FoxP3 led to decreased levels of TGF‐β and IL‐6 (*p* < .05, Figure 5D). The results of Transwell assays unveiled that the silencing of FoxP3 inhibited HCCA cell migration and invasion (*p* < .05, Figure 5E,F). The above findings suggested that the silencing of FoxP3 neutralizes the role of FoxM1 overexpression in promoting Treg cell immunosuppression and facilitating HCCA cell immune escape.

### 3.6 FoxM1 promotes FoxP3 transcription to recruit Treg cells and induce HCCA cell immune escape

**Figure 6:**
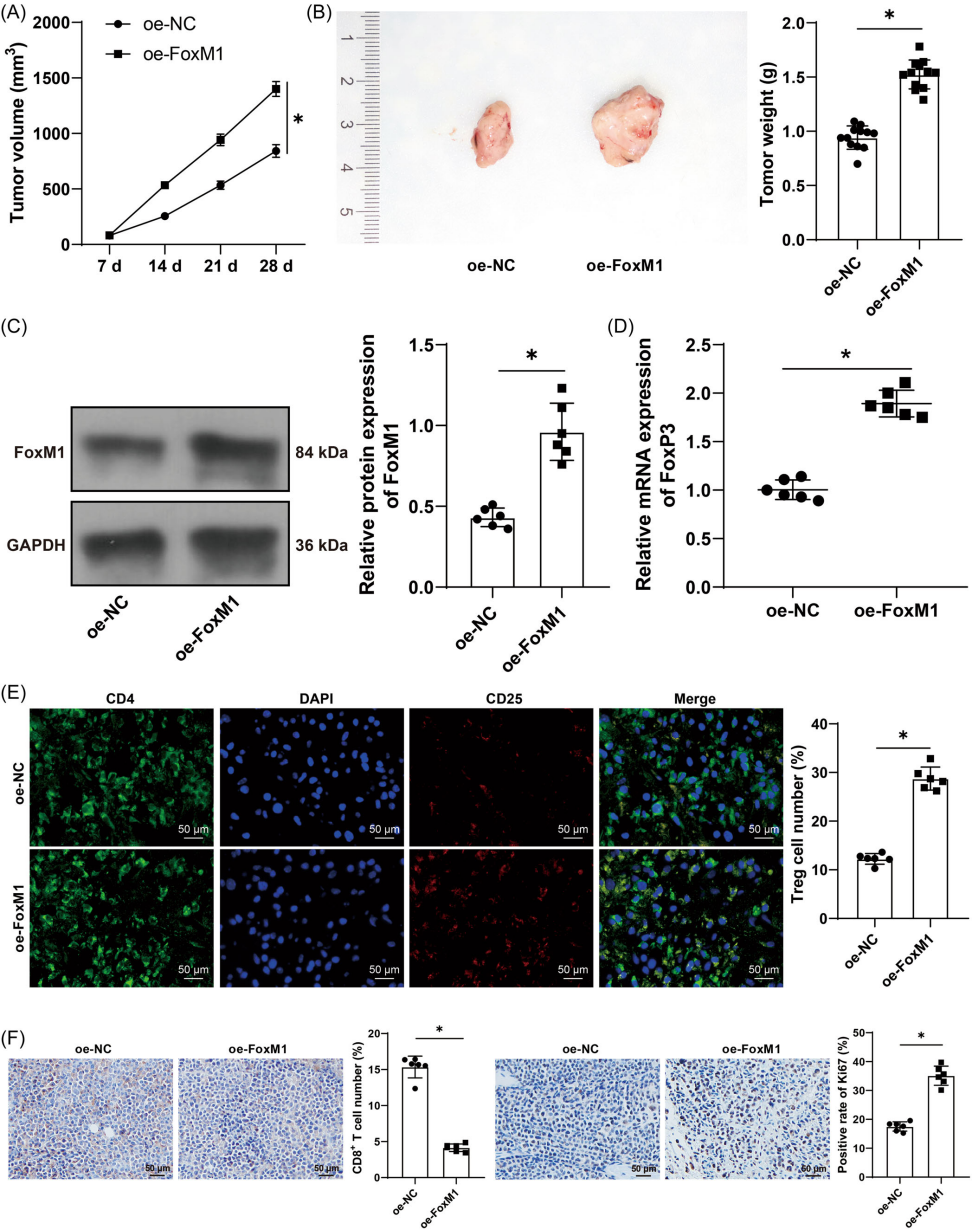
FoxM1 promotes FoxP3 transcription to recruit Treg cells, so as to induce HCCA cell immune escape. Xenografts tumor model was established in vivo as RBE cells with FoxM1 overexpression were subcutaneously injected into nude mice, with RBE cells with oe‐NC as the control. (A) and (B), volume (A) and weight (B) of tumors were examined (*N* = 12). (C) FoxM1 protein level was determined by Western blot assay. (D) FoxP3 mRNA level was determined by RT‐qPCR (*N* = 6). (E) the number of CD4^+^ CD25^+^ Treg cells was measured by dual enzyme‐labeled immunofluorescence (*N* = 6). (F) the number of CD8^+^ T cells and Ki67 level were tested by IHC (*N* = 6). The results were expressed as mean ± standard deviation. Two‐way analysis of variance was appointed to analyze the data in (A), and the independent *t*‐test was used to analyze the data in (B), (C), (D), (E), and (F). Tukey's post‐hoc test was applied for the post‐hoc test. *p < .05. FoxM1, Forkhead box M1; RT‐qPCR, reverse transcription‐quantitative polymerase chain reaction.

Moreover, the xenografts tumor model was established in vivo by subcutaneously injecting RBE cells with FoxM1 overexpression into nude mice, after which tumor volume and weight were aggrandized (*p* < .05, Figure 6A,B). The protein levels of FoxM1 in tumors were increased (*p* < .05, Figure 6C). FoxP3 mRNA level in the xenograft tumors was detected through RT‐qPCR, which revealed that FoxP3 mRNA level was increased upon FoxM1 overexpression (*p* < .05, Figure 6D). Furthermore, dual enzyme‐labeled immunofluorescence was performed for the number of CD4^+^ CD8^+^ Treg cells, and the results suggested that the number of CD4^+^ CD25^+^ Treg cells was elevated upon FoxM1 overexpression (*p* < .05, Figure 6E). Subsequently, the results of IHC found that when FoxM1 was overexpressed, the number of CD8^+^ T was declined, and Ki67‐positive rate was increased (*p* < .05, Figure 6F), indicating that FoxM1 promoted FoxP3 transcription to recruit Treg cells, so as to induce HCCA cell immune escape.

## 4 DISCUSSION

HCCA is a life‐threatening tumor that is mainly diagnosed at the advanced stage. HCCA could be treated by resection, which might bring about severe postoperative sequelae.^22^ Immune cells exert cytotoxic effects to eliminate cancer cells in the early phase, but cancer cells with immune escape could antagonize immune cells.^23^ As a kind of oncogene, FoxM1 is robustly expressed in various human neoplasms and mediates target cytokine expression.^24^ Notably, FoxM1 is linked to cancer cell viability, dissemination, death, and immune escape.^25^ Based on the above information, we attempted to discuss the specific effect of FoxM1 on HCCA immune escape.

Immune escape plays a significant role in cancer surveillance, malignancy, and alleviation.^26^ CD8^+^ T cells are identified as a prospective option for cancer immunotherapies for their potent killing ability.^27^ To figure out the immune escape mechanism of HCCA cells, CD8^+^ T cells were co‐cultured with HCCA cells, and in the co‐culture system, the killing on HCCA cells by CD8^+^ T cells could be reversed by Treg cells. However, the immunologic function of CD8^+^ T cells can be greatly reduced by Treg cells, which formed an obstruction to enhancing immunosuppression and immune escape.^28^ Treg cells activate intrahepatic CC malignant reactions by enhancing immune escape.^29^ Collectively, Treg cells suppressed CD8^+^ T killing and enhanced HCCA cell proliferation.

FOXO family is a promising group of proteins to research the progression and treatment of carcinomas.^30^ FoxM1 expression was increased in intrahepatic CCA and associated with cancer cell growth and aggressiveness as well as poor prognostic consequences.^31^ In our study, FoxM1 was overexpressed in HCCA. FoxM1 intensified HCCA tumor expansion and led to a disappointing prognosis,^32^ which is in line with our findings. To elucidate the role of FoxM1 in HCCA cell immune escape, we overexpressed FoxM1 and found that FoxM1 overexpression attenuated CD8^+^ T killing on HCCA cells, increased the levels of TGF‐β and IL‐6, and enhanced HCCA cell migration and invasion in case of adding Treg cells. FoxM1 cooperated with Treg cells contributing to the pathological manifestation, immune escape, and unsatisfactory prognosis.^33^ FoxM1 depletion in hepatocellular carcinoma quenched carcinoma mobility and triggered immunoreaction by activating specific CD8^+^ T killing.^34^ Essentially, FoxM1 overexpression in CCA limited CD8^+^ T killing to promote cancer cell migration and defense against immunotherapeutic efficiency.^35^ Similarly, FoxM1 overexpression was related to increased TGF‐β levels, enhanced cellular resistance to drugs, and sabotaged immunoreaction in gastric carcinoma.^36^ The above findings suggested that FoxM1 overexpression accelerated immune escape of HCCA cells by promoting Treg cell immunosuppression on CD8^+^ T cells.

FoxP3 is a specific indicator of Treg cells and an essential inducer of immune escape to neoplasms.^37^ FoxP3 ablation contributed to reduced tumor invasiveness and immune escape in CCA.^38^ In this study, we found that FoxP3 was upregulated in HCCA cells and FoxM1 bound to the FoxP3 promoter region to promote FoxP3 transcription. Similarly, both FoxM1 and FoxP3 were strongly expressed in gastric cancer, resulting in tumor metastasis, immune escape, and poor prognosis.^21^ Our experiments revealed that the silencing of FoxP3 expression led to decreased levels of TGF‐β and IL‐6 as well as inhibited HCCA cell migration and invasion in case of adding Treg cells. The immunosuppressive and immune‐resistant properties of FoxP3^+^Treg cells could encourage immune escape, facilitate carcinoma growth, and reverse CD8^+^ T killing.^39^ A growing number of FoxP3^+^ Treg cells brought about bile duct invasiveness, balanced cancer microenvironment homeostasis, and a high chance of relapse in intrahepatic CCA.^40^ The absence of TGF‐β and IL‐2 was related to decreased expression of Foxp3 in Treg cells, thereby influencing different biological and pathological processes.^41^ The above findings suggested that the silencing of FoxP3 neutralizes the role of FoxM1 overexpression in promoting Treg cell immunosuppression and facilitating HCCA cell immune escape. Finally, the xenografts tumor model was established in vivo by injecting RBE cells with FoxM1 overexpression and we found that FoxM1 overexpression aggrandized tumor volume and weight, increased FoxP3 mRNA level the number of CD4^+^ CD25^+^ Treg cells and Ki67 level, but decreased the number of CD8^+^ T cells. The increased number of CD8^+^ T cells contributed to promoted cell death, restricted inflammatory symptoms, and upgraded cytotoxicity of CCA.^42^ Likewise, a previous report indicated that FoxM1 overexpression enhanced the number of CD4^+^CD8^+^ Treg cells and Ki67 protein levels, to strengthen the immune escape of gastric cancer.^43^ Our findings indicated that FoxM1 promoted FoxP3 transcription to recruit Treg cells, so as to induce HCCA cell immune escape.

## 5 CONCLUSION

In this study, our findings supported that FoxM1 bound to the FoxP3 promoter region to promote FoxP3 transcription and recruit FoxP3^+^ Treg cells, thereby inducing HCCA immune escape. These findings suggested a therapeutic strategy for HCCA alleviation. However, there are some limitations to our study. On the one hand, this study failed to conduct clinical validation and detect FoxP3 protein levels; on the other hand, the role of other members of the FOXO family in regulating FoxP3 in HCCA is unclear. Besides, we did not perform FoxM1 silencing assays as a complementary approach to these findings. In future research, the relation between FoxM1 and FoxP3^+^ Treg cells in specimens from HCCA patients will be investigated, changes in FoxP3 protein level in HCCA immune escape will be explored, and the role of other members of the FOXO family in regulating FoxP3 in HCCA will be probed.

## AUTHOR CONTRIBUTIONS

Kai Ma and Zhaowei Sun made substantial contributions to the conception of the present study. Xueliang LiL and Jingyun Guo performed the experiments and wrote the manuscript. Qinlei Wang and Mujian Teng contributed to the design of the present study and interpreted the data. All authors read and approved the final version of the manuscript for publication.

## CONFLICT OF INTEREST

The authors declare no conflict of interest.

## Data Availability

The data that support this study are available from the corresponding author upon reasonable request.
